# Patient Characteristics and Perspectives of Firearm Safety Discussions in the Emergency Department

**DOI:** 10.5811/westjem.2021.3.49333

**Published:** 2021-05-19

**Authors:** Lauren Hudak, Henry Schwimmer, William Warnock, Sarah Kilborn, Tim Moran, Jeremy Ackerman, Jonathan Rupp

**Affiliations:** * Emory University, Department of Emergency Medicine, Atlanta, Georgia; † Alameda Health System, Highland Hospital, Department of Emergency Medicine, Oakland, California; ‡ Emory University, School of Medicine, Atlanta, Georgia; § Vanderbilt University, Department of Emergency Medicine, Nashville, Tennessee

## Abstract

**Introduction:**

Firearm injury prevention discussions with emergency department (ED) patients provide a unique opportunity to prevent death and injury in high-risk patient groups. Building mutual understanding of safe firearm practices between patients and providers will aid the development of effective interventions. Examining ED patient baseline characteristics, perspectives on healthcare-based safety discussions, and experience with and access to firearms, will allow practitioners to craft more effective messaging and interventions.

**Methods:**

Using an institutional review board-approved cross-sectional survey modified from a validated national instrument, we recruited 625 patients from three large, urban, academically affiliated EDs in the South to assess patient baseline characteristics, perspectives regarding firearms and firearm safety discussions, and prior violence history, as well as firearm access and safety habits. We compared the degree to which patients were open to discussions regarding firearms across a variety of provider types and clinical scenarios between those with and without gun access.

**Results:**

Of the 625 patients consented and eligible for the study, 306 had access to firearms. The patients with firearm access were predominantly male, were more likely to have military experience, live in an urban or suburban region, and have experienced prior violence when compared to those without firearm access. Patients with and without gun access view firearm safety discussions with their healthcare provider as acceptable and analogous to other behavioral health interventions (i.e., helmet/seat belt use, alcohol/cigarette use). Patients were also accepting of these firearm safety discussions in many clinical contexts and led by multiple provider types. Of the patients with gun access, storage of each type of firearm was reviewed and the primary reason for ownership was for personal protection across all firearm types.

**Conclusion:**

Patients in the ED indicate openness to firearm safety discussions delivered by a variety of providers and in diverse clinical scenarios. Healthcare providers engaging firearm owners in appropriate risk-benefit discussions using a trauma-informed approach is a critical next step in research and intervention.

## INTRODUCTION

Although firearm injury is widely recognized as a public health epidemic responsible for approximately 40,000 deaths and 130,000 injuries in the United States in 2017 alone, the field has a dearth of rigorous research to guide effective intervention strategies.^1^ Additionally, there is limited research addressing firearm injury prevention in the healthcare setting, likely contributing to a lack of engagement and general discomfort with the subject among patients and providers. Despite healthcare providers and medical societies advocating for firearm injury risk and safety discussions with patients,^2^ a minority of providers report initiating these conversations.^3^ Given concern for rising numbers of violence-related injuries,^4^ increased social isolation, and prevalence of mental health problems,^5^,^6^ as well as escalating firearm and ammunition purchases during the COVID-19 pandemic,^7^,^8^ these discussions are more critical now than ever. In fact, physicians and other healthcare providers are uniquely positioned to address this issue, as other potential avenues for intervention are limited due to social distancing and other lockdown measures.

Prior studies have touched on important elements to consider when addressing firearm safety in healthcare populations. The 2015 National Firearm Survey (NFS) used a nationally representative, web-based sample to estimate that 54.7 million people in the US own guns.^9^ Additionally, two-thirds of non-firearm owners and over one-half of firearm owners felt it is “at least sometimes appropriate” for physicians and other healthcare providers to discuss firearm safety with patients.^10^ Another study using the NFS sample examined the responses of veterans. They concluded that half of veterans own at least one firearm, with the majority owning both handguns and long guns, citing personal protection as the primary reason for ownership.^11^ These findings provide an important glimpse into firearm ownership and potential translational healthcare applications. However, the NFS was not designed solely for healthcare-based intervention and thus did not sample from patients in a clinical environment and did not expand upon potentially relevant healthcare-focused variables. Assessing patients’ degree of openness to firearm discussions with different healthcare provider types in specific clinical scenarios is an important next step in firearm injury prevention research.

Another study of 200 ED patients that used a 22-item survey to assess patient demographics, access to firearms, and general attitude toward healthcare-based screening comes closer to understanding ED patients’ views on firearm safety discussions. Their findings indicate the majority of both gun owning (100%) and non-owning patients (87.5%) felt comfortable discussing firearm safety with their healthcare provider, and a majority of patients felt these discussions would result in safer firearm storage changes.^12^ The patients’ views of different provider types conducting firearm safety discussions and clinical scenarios in which safety discussions are appropriate was not reported. Neither patients’ history of violence nor reasons for gun ownership were reported.

**Population Health Research Capsule**

What do we already know about this issue?

*Healthcare providers engaging patients in firearm safety discussions is emerging as a promising opportunity to prevent associated firearm injury and death.*

What was the research question?

*What are the characteristics of patients and in which clinical scenarios are firearm safety discussions acceptable?*

What was the major finding of the study?

*ED patients are open to firearm safety discussions delivered by a variety of providers and in diverse clinical scenarios.*

How does this improve population health?

*Healthcare providers can engage patients in firearm safety discussions with the goal of reducing risk for firearm injury and death.*

More broadly, healthcare interventions that involve firearm safety or storage counseling, such as lethal means counseling, have become established as effective in healthcare populations, especially in suicidal adult and pediatric mental health populations.^13^,^14^ These interventions have gained traction in ED settings,^3^,^15^–^17^ with a focus on providers building knowledge about firearms and safety practices in an effort to build cultural competence to better engage gun owners in safety discussions and primary prevention.^18^ Such efforts have improved our understanding of healthcare-focused safety discussions. Further exploring the factors that contribute to ED patient attitudes and potential receptivity to intervention is critical to advancing the field and saving lives.

This cross-sectional study addresses these gaps in understanding by surveying the attitudes and experiences of ED patients. The knowledge gained directly contributes to the development of effective intervention with ED patients by evaluating their baseline demographics, firearm-related discussion perspectives, prior experience of violence, and firearm access and safety practices.

## METHODS

After institutional review board approval, registered ED patients were approached by trained research assistants (RA) during convenience sample shifts from 7 am–7 pm, seven days per week in three academically affiliated urban EDs in Atlanta, Georgia, from October 2018–April 2019. The largest hospital, with annual ED visit volume of approximately 142,000, is a Level I trauma center serving mainly an urban, largely underinsured population. The second hospital, with approximately 74,000 annual ED visits, also serves an urban patient population as a community-affiliated academic medical center. The third ED, a tertiary medical center on an academic campus has approximately 51,000 annual visits. Eligible patients were those who did not meet exclusion criteria (<18 years of age, non-English literate, cognitively impaired, medically unstable, in police custody, had previously participated) and from whom verbal informed consent was obtained prior to enrollment. Survey instruments were administered using Apple iPads (Apple, Inc., Cupertino, CA) and REDCap, a web-based software program compliant with the Healthcare Insurance Portability and Accountability Act of 1996. Question types included five-point Likert-type, multiple choice, binary yes/no, and free-text responses, and questions were presented only when relevant to the patient using branching logic (up to 198 questions). After providing consent, the RAs instructed patients on self-administration of the survey using the tablet computers. Patients who declined participation were asked a reason for their decision, and if provided, the RA recorded their response in the free-text portion of the approach section.

### Survey Domains

The survey is divided into three domain areas: 1) demographic information; 2) firearm-related perspectives and past experiences; and 3) firearm access and safety habits. Participants were not permitted to return to prior forms when the domain was completed. Demographic variables of interest included age, gender, race, ethnicity, marital status, housing type/region, education, employment status, income, number of children/if housing them, and military status.

The firearm-related perspectives domain contained a wide range of potentially relevant firearm-related attitudes and experiences as well as topics considered important for potential intervention. Less invasive topics were explored first, such as general perspectives on health-related issues, escalating to potentially more invasive topics, such as political views and prior experience of violence. Public health context of firearm discussions relative to other clinical safety discussions, acceptability of different provider types, acceptability of discussing firearm safety in different clinical scenarios, as well as prior violence history were assessed for this phase of the study. For complete survey elements please reference the supplement section.

The firearm access domain ushered participants through a branching logic survey tool to establish current firearm access and safety habits. Firearm “access” is the preferred terminology for the purposes of this study, as it is a more inclusive term compared to personal “ownership,” acknowledging the potential for fluid possession in households or other unforeseeable shared-use situations. To capture the relevant possibilities of firearm access, subjects were asked, “Do you or does anyone else you live with currently own any type of gun?” and “What type of gun do you own or have access to?” Additionally, the term firearm and gun are used interchangeably for the purposes of this study, with acknowledgment that the term firearm is more inclusive. We obtained detailed assessment of the reason(s) for ownership and location of the firearm(s), as well as storage habit(s) for each firearm.

Firearms were subdivided into handguns, long guns and “other” guns; storage habits and locations were reviewed for each firearm. Handguns include pistols, revolvers, semi-automatic pistols/revolvers, and “other” as designated by the participant. Long guns include shotguns, rifles, modern sporting rifles, and “other” as designated by the participant. Free space was allowed for the patient to elaborate on any “other type of gun” to which they had access. Survey methodology was conducted in alignment with the question types and terminology used in the 2015 National Firearm Survey and validated by independent expert consensus.

### Statistical Analyses

We described continuous variables using medians and interquartile ranges. Categorical variables were described using frequencies and percentages. We compared patient demographics across those with gun access and those without gun access using the Mann-Whitney U test and the χ^2^ test for continuous and categorical variables, respectively. The main outcomes of interest – patient comfort with questions regarding gun access – were compared across groups using separate ordinal logistic, generalized estimating equations for each provider type. We used the generalized estimating equation to account for clustering within hospital. The adjusted regression included age, gender, race, ethnicity, marital status, region, housing, education, income, number of children, and military experience as covariates. Odds ratios and 95% confidence intervals from the analyses are presented. Analyses were conducted using SPSS v.25 (IBM Corporation, Armonk, NY)

## RESULTS

Of the 1482 patients approached by RAs for inclusion in the study, 625 were eligible and consented to participate. Of those patients, 306 patients had access to firearms while 319 did not. A total of 733 patients declined to participate with various reasons provided in a qualitative free-text response. Other than medical/pain-related concerns, patients cited being tired (n = 97), that the survey was anticipated to take too long (n = 41), or they had already been approached/taken survey (n = 13) as common reasons for non-participation. Additionally, some patients declined due to discomfort with firearms as the survey topic (n = 41), or dislike of firearms (n = 16), or they declined due to some other discomfort with the topic of firearms (n = 25).

### Demographics

When comparing those without firearm access to those with access a few key features emerged (Table 1). Study patients with firearm access (n = 191, 62.4%) were more likely to be male when compared to those without access (n = 135, 42.3%). Black participants formed the majority of both groups (+access n = 176, 57.5%; −access n = 221, 69.3%), but our gun-accessing population self-identified more frequently as White (n = 85, 27.8%) when compared to the no access group (n = 66, 20.7%). Those with firearm access tended to report being married (n = 98, 32.0%) and home-dwelling (n = 184, 60.1%) more often when compared to the non-firearm accessing group (n = 58, 18.2% and n = 151, 47.3%, respectively). The majority of non-firearm accessing individuals reported living in an urban environment (n = 180, 56.4%) in comparison to those with access (n = 120, 39.2%), who were more likely to live in suburban (n = 123, 40.2%) or rural (n = 63, 20.6%) regions. There was no significant difference between education and employment levels in our population, although patients with firearm access were more affluent and had fewer children than the non-access patients. Those with firearm access were also more likely to have military experience (n = 42, 13.7%) than the non-access (n = 17, 5.3%) group.

### Perspectives

#### Firearm Discussions Compared to Other Behavioral Health Discussions

We reviewed patient opinion regarding the acceptability of firearm-safety discussions relative to analogous behavioral health topics. Patients generally agreed that firearms should be regarded similarly to other public health topics, such as cigarette smoking, alcohol use, and use of helmets and seatbelts. While agreement was high for both those with and without firearm access, those with access agreed to a lesser extent than their non-accessing counterparts (Table 2).

#### Firearm Discussions Comparing Healthcare Provider Types

**Figure 1:**
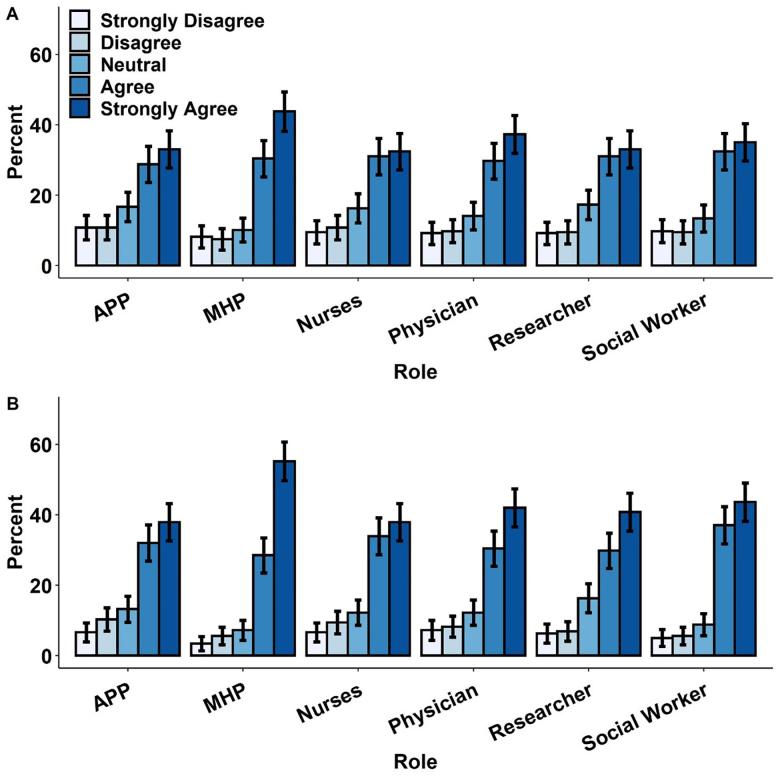
Patient degree of agreement that discussing with each provider type is appropriate in A (patients with gun access) and B (patients without gun access). Error bars represent 95% confidence intervals. *APP*, advanced practice providers; *MHP*, mental health professional.

As in prior studies, it appears both groups were in agreement that asking about firearms is appropriate. Patients with gun access were less likely to strongly agree that it is appropriate for providers to conduct medically indicated firearm safety discussions compared with patients without access, although they still generally found such discussions acceptable. Of note, both patients with gun access and those without access agreed that it was most appropriate to have gun safety discussions with mental health providers followed by physicians, while discussions with nurses and researchers were marginally less appropriate but still acceptable overall. (Table 3, Figure 1).

#### Firearm Discussions in Various Clinical Scenarios

Patients were generally in agreement that it is appropriate to discuss firearm risk/safety across multiple clinical scenarios. Both the firearm access and no access groups agreed (*P*-value <.001) that providers can ask about firearms in the following clinical scenarios: personal and family depressed/suffering from mental health issues; children in the home; personal or family memory problems; cases of suspected domestic violence; and victim or perpetrator of violent injury. As with the provider type, while both patients with and without access to firearms generally believed it was appropriate to discuss firearms in these contexts, agreement was lower for those with access (Table 4).

#### Patient Past Experience of Violence

Past experience of violence was highly prevalent for both those with and without access to firearms. Notably, those with access to firearms experienced significantly more workplace violence (n = 70, 22.9%) and had been shot (n = 62, 20.3%) significantly more than those with no access (n = 22, 6.9% and n = 23, 7.2% respectively). Additionally, those with access were more likely to report having been “pistol whipped” or struck with a gun (n =56, 18% vs n = 28, 8.8%), unintentionally shooting themselves or others (n = 56, 18% vs n=28, 8.8%), and reporting medical treatment due to firearm-related injury (n = 73, 23.9% vs n = 11, 3.4%) than those without access. Other types of violence such as physical violence, sexual violence, and domestic violence, while prevalent, did not differ significantly between groups (Table 5).

### Access

Gun-accessing patients made up about half of the sample with 306 of 625 participants total having access to firearms. Of the handguns reviewed, 19.1% of patients indicated that they stored them “loaded and unlocked,” which is regarded as the least safe of possible options. Long guns followed a similar pattern with 19.3% of patients storing them “loaded and unlocked.” Conversely, 31.9% of patients’ handguns and 33.3% of patients’ long guns were designated as “unloaded and locked,” which is regarded as the safest of possible options. Of patients’ “other guns” category, 29.7% of patients stored them “loaded and unlocked,” while 23.1% stored them “unloaded and locked” (Table 6).

Patients with firearm access indicated that their primary reason for ownership was for “personal protection” inclusive of protection against both “strangers” and “people I know.” Handguns were owned for “personal protection” (84.3%) followed distantly by “hunting” (23.4%), “other sporting use” (15.7%), “some other reason” (14.0%), and “collection/hobby” (13.2%). For long guns, “personal protection” still led (67%) with “hunting” by a closer margin (46%), and “other sporting use” (28.0%), “collection/hobby” (26.0%), and “some other reason” (9.3%) following thereafter. Other guns were owned for “personal protection” in the majority of cases (67.0%) followed by “some other reason” (33.0%), “other sporting use” (12.1%), and “collection/hobby” (11.0%).

## DISCUSSION

Firearm injury prevention and safety discussions in the healthcare setting are emerging as promising intervention opportunities to reduce injury burden on communities. By surveying patients in three clinically diverse ED populations, we sought to better understand the motivations, attitudes, and experiences of patients likely to be the focus of future safety intervention. The degree of firearm ownership with various demographic groups tends to mirror national estimates, with a large proportion of gun-accessing patients being male with prior military service, but a higher degree of patients self-identifying as Black, living in an urban or suburban region in this particular sample. Consistent with prior studies, patients reported being open to firearm discussions with their doctor or healthcare provider, suggesting support for potential clinical interventions.

In this study, patients generally regarded firearm safety discussions as similar to other clinically relevant topics such as helmet use, seatbelt wearing, and substance use counseling. Furthermore, novel findings support that patients (both firearm accessing and not) find firearm safety discussions acceptable and appropriate in a wide variety of clinical scenarios and coming from diverse healthcare provider types, which has not been explored in prior research settings. Somewhat surprisingly, the investigators found a very high prevalence of violent victimization in the study population. The number of firearm-accessing patients who had been shot, pistol whipped, or had accidentally shot themselves or others merits further analysis and research attention. Patients claim personal protection as their primary reason for ownership across all firearm types, which has implications for future intervention counseling, especially when considering the potential for history of violent victimization. Handguns, the firearm type most associated with self-inflicted and interpersonal violence,^19^ were not stored in the safest manner, “unloaded and locked,” providing potential room for further exploration and intervention in this high-risk population.

The results presented here lend investigators a more informed perspective when approaching firearm safety discussions in a largely urban population with a high prevalence of violence. By tailoring risk-benefit and safety counseling discussions to local customs, norms, and attitudes, future interventions can be pursued using a regionally relevant, evidence-based framework. Additionally, the findings here support the growing body of evidence calling for interventions that emphasize a trauma-informed approach^20^ to ensure future intervention approaches recognize the impact of past violence on patient attitude, behavior, and health.

## LIMITATIONS

There are multiple limitations when interpreting the results of this study. Patients were recruited from three clinically diverse urban, southern EDs, with a large proportion self-identifying as Black and lower income, with a high prevalence of violent victimization. The results may not be generalizable to other regions or different demographic groups. Additionally, the inherent nature of survey-based methodology introduces the potential for sampling bias, participant response bias, and question-order bias. Efforts to reduce the effects of these biases were made in constructing the survey based on prior national, validated survey instruments and validating the new survey instrument through extensive piloting and expert review. The ability to lock each survey domain was used in an effort to limit participant response bias, especially with respect to the perspectives and access survey-domain responses.

Another limitation of the study was survey length. In particular, the firearm-accessing respondents had the potential to receive up to 198 questions. Efforts to reduce survey length were created by using branching logic question templates to reduce unnecessary questioning and tailor questions specific to the respondent. Unfortunately, the survey length could have resulted in answer fatigue and bias in survey responses. Encouragement prompts were used in the survey instrument in an effort to pace participants, as were RAs trained to assist if interruptions occurred. The extensive questioning also poses its own limitation in that the vast amount of data for potential review limited the ability to present all interesting and potentially relevant findings and will require subsequent analyses to further explore the population nuances in future research.

## CONCLUSION

Firearm safety discussions in the ED are well accepted by patients and can be delivered by a variety of providers in diverse clinical scenarios. This concept builds upon research supporting such safety discussions in healthcare populations, despite perceived potential discomfort experienced by both providers and patients. Engaging firearm owners in respectful, culturally appropriate risk-benefit discussions with trained providers offers a promising opportunity to improve safety and storage habits in high-risk populations. Furthermore, using a trauma-informed approach, especially considering patient past experience of violence, should be considered and further explored in future research.

## Supplementary Information





## Figures and Tables

**Table 1:** Demographic characteristics of study participants, gun access vs no access.

Characteristic	No access N = 319	Gun access N = 306	Total N = 625	P-value
Age	45 (30 – 56.5)	47.5 (34 – 61)		0.01
Gender				< .001
Female	184 (57.7)	115 (37.6)	299	
Male	135 (42.3)	191 (62.4)	326	
Race				0.02
American Indian/Alaska Native	4 (1.3)	7 (2.3)	11	
Asian	9 (2.8)	6 (2)	15	
Black	221 (69.3)	176 (57.5)	397	
Multiple	15 (4.7)	23 (7.5)	38	
Native Hawaiian	4 (1.3)	9 (2.9)	13	
White	66 (20.7)	85 (27.8)	151	
Ethnicity				0.38
Not Hispanic	296 (92.8)	283 (92.5)	579	
Hispanic	23 (7.2)	23 (7.5)	46	
Marital status				0.002
Divorced	47 (14.7)	45 (14.7)	92	
Married	58 (18.2)	98 (32)	156	
Unmarried couple	25 (7.8)	26 (8.5)	51	
Separated	20 (6.3)	11 (3.6)	31	
Single	156 (48.9)	112 (36.6)	268	
Widowed	13 (4.1)	14 (4.6)	27	
Housing				0.004
Apartment	126 (39.5)	74 (24.2)	200	
House	151 (47.3)	184 (60.1)	335	
Homeless	10 (3.1)	9 (2.9)	19	
Hotel	4 (1.3)	4 (1.3)	8	
Nursing home/assisted living	4 (1.3)	10 (3.3)	14	
Shelter	6 (1.9)	7 (2.3)	13	
Staying with friends/family	18 (5.6)	18 (5.9)	36	
Region				< .001
Rural	26 (8.2)	63 (20.6)	89	
Suburban	113 (35.4)	123 (40.2)	236	
Urban	180 (56.4)	120 (39.2)	300	
Highest education level				0.11
No school or only kindergarten	2 (0.6)	9 (2.9)	11	
Elementary	4 (1.3)	7 (2.3)	11	
Some high school	38 (11.9)	29 (9.5)	67	
High School graduate or GED	105 (32.9)	84 (27.5)	189	
Some college or technical school	101 (31.7)	99 (32.4)	200	
College graduate	69 (21.6)	78 (25.5)	147	
Employment				0.06
Homemaker	16 (5)	11 (3.6)	27	
Student	32 (10)	20 (6.5)	52	
Employed	127 (39.8)	126 (41.2)	253	
Employment				0.06
Out of work (<1 year)	25 (7.8)	19 (6.2)	44	
Out of work (>1 year)	47 (14.7)	35 (11.4)	82	
Retired	50 (15.7)	55 (18)	105	
Self-employed	22 (6.9)	42 (13.7)	64	
Income				0.01
<$15,000	114 (35.7)	77 (25.2)	191	
$15,000–25,000	58 (18.2)	45 (14.7)	103	
$25,000–35,000	35 (11)	36 (11.8)	71	
$35,000–50,000	33 (10.3)	50 (16.3)	83	
$50,000–75,000	37 (11.6)	38 (12.4)	75	
<$75,000	42 (13.2)	60 (19.6)	102	
Number of children				0.02
0	172 (53.9)	125 (40.8)	297	
1	41 (12.9)	44 (14.4)	85	
2	51 (16)	58 (19)	109	
3	25 (7.8)	31 (10.1)	56	
4+	30 (9.4)	48 (15.7)	78	
Number of children in home				0.9
0	206 (64.6)	191 (62.4)	397	
1	49 (15.4)	48 (15.7)	97	
2	33 (10.3)	33 (10.8)	66	
3	15 (4.7)	15 (4.9)	30	
4+	16 (5)	19 (6.2)	35	
Military experience, N (%)	17 (5.3)	42 (13.7)	59	0.001

**Table 2:** Opinion of study patients on discussing different public health topics with a provider: gun access vs no gun access.

Public health topic	Strongly disagree	Disagree	Neither agree nor disagree	Agree	Strongly agree	OR (95% CI, unadjusted)	OR (95% CI, adjusted)
Smoking cigarettes						0.44 (0.31 – 0.64)	0.45 (0.31 – 0.65)
No access	22 (6.9)	13 (4.1)	8 (2.5)	77 (24.1)	197 (61.8)		
Gun access	40 (13.1)	33 (10.8)	29 (9.5)	70 (22.9)	134 (43.8)		
Drinking alcohol						0.48 (0.32 – 0.72)	0.48 (0.32 – 0.72)
No access	24 (7.5)	14 (4.4)	10 (3.1)	74 (23.2)	197 (61.8)		
Gun access	32 (10.5)	37 (12.1)	26 (8.5)	72 (23.5)	139 (45.4)		
Helmet use						0.60 (0.40 – 0.90)	0.67 (0.44 – 1.02)
No access	18 (5.6)	21 (6.6)	43 (13.5)	91 (28.5)	146 (45.8)		
Gun access	33 (10.8)	42 (13.7)	46 (15)	71 (23.2)	114 (37.3)		
Seatbelt use						0.46 (0.33 – 0.66)	0.52 (0.36 – 0.75)
No access	12 (3.8)	15 (4.7)	30 (9.4)	91 (28.5)	171 (53.6)		
Gun access	39 (12.7)	41 (13.4)	39 (12.7)	65 (21.2)	122 (39.9)		
Gun safety						0.57 (0.4 – 0.79)	0.60 (0.41 – 0.88)
No access	33 (10.3)	27 (8.5)	34 (10.7)	82 (25.7)	143 (44.8)		
Gun access	44 (14.4)	45 (14.7)	46 (15)	71 (23.2)	100 (32.7)		

*OR*, odds ratio; *CI*, confidence interval.

**Table 3:** Opinion of patients regarding provider type initiating firearm safety discussions, gun access vs no gun access.

Provider type that can ask about gun access if medically indicated	Strongly disagree	Disagree	Neither agree nor disagree	Agree	Strongly agree	OR (95% CI, unadjusted)	OR (95% CI, adjusted)
Physician						0.81 (0.59 – 1.09)	0.98 (0.67 – 1.42)
No access	23 (7.2)	26 (8.1)	39 (12.2)	97 (30.4)	134 (42.0)		
Gun access	28 (9.2)	30 (9.8)	43 (14.1)	91 (29.7)	114 (37.3)		
APP						0.74 (0.53 – 1.02)	0.85 (0.59 – 1.22)
No access	21 (6.6)	33 (10.3)	42 (13.2)	102 (32.0)	121 (37.9)		
Gun access	33 (10.8)	33 (10.8)	51 (16.7)	88 (28.8)	101 (33.0)		
Nurse						0.73 (0.54 – 0.99)	0.82 (0.57 – 1.19)
No access	21 (6.6)	30 (9.4)	39 (12.2)	108 (33.9)	121 (37.9)		
Gun access	29 (9.5)	33 (10.8)	50 (16.3)	95 (31.0)	99 (32.4)		
Social Worker						0.61 (0.44 – 0.86)	0.67 (0.45 – 0.99)
No access	16 (5.0)	18 (5.6)	28 (8.8)	118 (37.0)	139 (43.6)		
Gun access	30 (9.8)	29 (9.5)	41 (13.4)	99 (32.4)	107 (35.0)		
MHP						0.60 (0.42 – 0.86)	0.73 (0.49 – 1.09)
No access	11 (3.4)	18 (5.6)	23 (7.2)	91 (28.5)	176 (55.2)		
Gun access	25 (8.2)	23 (7.5)	31 (10.1)	93 (30.4)	134 (43.8)		
Researchers						0.72 (0.51 – 1.01)	0.76 (0.51 – 1.13)
No access	20 (6.3)	22 (6.9)	52 (16.3)	95 (29.8)	130 (40.8)		
Gun access	28 (9.2)	29 (9.5)	53 (17.3)	95 (31.0)	101 (33.0)		

*APP*, advanced practice provider, *MHP*, mental health provider, *OR*, odds ratio, *CI*, confidence interval.

**Table 4:** Opinion of patients on providers asking about access to guns in various clinical settings, gun access vs no gun access.

It is ok for providers to ask patient about access to guns	Strongly Disagree, N (%)	Disagree, N (%)	Neither Agree nor Disagree, N (%)	Agree, N (%)	Strongly Agree, N (%)	P-value
If depressed/suffering from mental health						< .001
No access	22 (6.9)	21 (6.6)	15 (4.7)	73 (22.9)	188 (58.9)	
Gun access	39 (12.7)	36 (11.8)	39 (12.7)	65 (21.2)	127 (41.5)	
If family depressed/suffering from mental health						< .001
No access	20 (6.3)	22 (6.9)	18 (5.6)	76 (23.8)	183 (57.4)	
Gun access	38 (12.4)	43 (14.1)	44 (14.4)	62 (20.3)	119 (38.9)	
If there are children in the home						< .001
No access	19 (6)	17 (5.3)	31 (9.7)	72 (22.6)	180 (56.4)	
Gun access	43 (14.1)	40 (13.1)	39 (12.7)	63 (20.6)	121 (39.5)	
If I am elderly/have memory problems						< .001
No access	21 (6.6)	22 (6.9)	33 (10.3)	68 (21.3)	175 (54.9)	
Gun access	44 (14.4)	40 (13.1)	48 (15.7)	54 (17.6)	120 (39.2)	
If family member is elderly/has memory problems						< .001
No access	20 (6.3)	29 (9.1)	36 (11.3)	63 (19.7)	171 (53.6)	
Gun access	37 (12.1)	48 (15.7)	45 (14.7)	66 (21.6)	110 (35.9)	
In cases of suspected domestic violence						< .001
No access	22 (6.9)	20 (6.3)	20 (6.3)	60 (18.8)	197 (61.8)	
Gun access	41 (13.4)	42 (13.7)	38 (12.4)	58 (19)	127 (41.5)	
If I am the victim of violent injury						< .001
No access	20 (6.3)	24 (7.5)	22 (6.9)	73 (22.9)	180 (56.4)	
Gun access	37 (12.1)	44 (14.4)	43 (14.1)	58 (19)	124 (40.5)	
If I am the perpetrator of violent injury						< .001
No access	21 (6.6)	23 (7.2)	22 (6.9)	61 (19.1)	192 (60.2)	
Gun access	35 (11.4)	33 (10.8)	49 (16)	59 (19.3)	130 (42.5)	

P-values were computed using the x^2^ test.

**Table 5:** History of violence among study patients, gun access vs no gun access.

Violent experience type, N (%)	No gun access	Gun access	P-value
Victim of physical violence	103 (32.3)	121 (39.5)	0.1
Was a gun used?	36 (35)	48 (39.7)	0.56
Victim of sexual violence	54 (16.9)	68 (22.2)	0.21
Was a gun used?	13 (24.1)	25 (36.8)	0.19
Victim of domestic violence	77 (24.1)	86 (28.1)	0.36
Was a gun used?	13 (16.9)	27 (31.4)	0.049
Workplace violence	22 (6.9)	70 (22.9)	0.01
Was a gun used?	6 (27.3)	22 (31.4)	0.92
Been shot	23 (7.2)	62 (20.3)	0.01
Been struck/pistol whipped	28 (8.8)	56 (18.3)	0.01
Accidentally shot self/others	8 (2.5)	42 (13.7)	< .001
Needed medical treatment	11 (3.4)	73 (23.9)	< .001
Other injury after threatened by gun	21 (6.6)	41 (13.4)	0.047
Gang affiliation	10 (3.1)	3 (1)	0.11

P-values were computed using the x^2^ test.

**Table 6:** Patient primary reason for gun ownership and gun storage method.

	Handgun	Long gun	Other gun
Reason for owning, N (%)			
Hunting	55 (23.4)	69 (46)	0 (0)
Personal protection	198 (84.3)	92 (61.3)	61 (67)
Collection/hobby	31 (13.2)	39 (26)	10 (11)
Other sporting use	37 (15.7)	42 (28)	11 (12.1)
Some other reason	33 (14)	14 (9.3)	30 (33)
Storage method, N (%)			
Loaded and unlocked	45 (19.1)	29 (19.3)	27 (29.7)
Unloaded and unlocked	75 (31.9)	33 (22)	18 (19.8)
Loaded and locked	75 (31.9)	39 (26)	25 (27.5)
Unloaded and locked	40 (17)	50 (33.3)	21 (23.1)
