# Supplementary material for: Patient Characteristics and Perspectives of Firearm Safety Discussions in the Emergency Department
Source: West J Emerg Med. 2021 May 19;22(3):478–87. doi: 10.5811/westjem.2021.3.49333 (PMC8203031; doi:10.5811/westjem.2021.3.49333)
Supplement: Supplementary file 1 [file wjem-22-478-s001.pdf]

Perspectives

Throughout this survey we use the word gun to refer to any firearm, including pistols, revolvers, shotguns and rifles, but not including air guns, bb guns, starter pistols or paintball guns. For the purposes of the survey, the terms gun and firearm are used interchangeably.

You have completed 25% of the survey.

If medically indicated, it is okay for healthcare providers to ask their patients about:

	Strongly agree	Agree	Neither agree nor disagree	Disagree	Strongly disagree
cigarette smoking	○	○	○	○	○
alcohol use	○	○	○	○	○
helmet use	○	○	○	○	○
seat belt use	○	○	○	○	○
access to guns	○	○	○	○	○

If medically indicated, it is okay for the following types of healthcare providers to ask patients about access to guns:

	Strongly agree	Agree	Neither agree nor disagree	Disagree	Strongly disagree
Physicians	○	○	○	○	○
Advanced Practice Providers (Physician Assistants, Nurse Practitioners)	○	○	○	○	○
Nurses	○	○	○	○	○
Social Workers	○	○	○	○	○
Mental Health Providers	○	○	○	○	○
Researchers	○	○	○	○	○

If you do not think it is okay to ask about guns in the healthcare setting, why not?

☐ Not relevant to my health
☐ Too private
☐ Don't want it recorded in the medical record
☐ Other
(select all that apply)

Other

____
(why?)

It is okay for providers to ask about ACCESS to guns...

	Strongly agree	Agree	Neither agree nor disagree	Disagree	Strongly disagree
if I am depressed, suicidal, or suffering from other mental health issues	○	○	○	○	○
if a family member is depressed, suicidal, or suffering from other mental health issues	○	○	○	○	○
if there are children in the home	○	○	○	○	○
if I am elderly with memory problems	○	○	○	○	○
if a family member is elderly with memory problems	○	○	○	○	○
in cases of suspected domestic violence	○	○	○	○	○
if I am the victim of a violent injury	○	○	○	○	○
if I am the perpetrator of violent injury	○	○	○	○	○

How strongly do you agree with the following?

	Strongly agree	Agree	Neither agree nor disagree	Disagree	Strongly disagree
Firearm injury is a public health problem.	○	○	○	○	○
Firearm safety is important to my health.	○	○	○	○	○
Firearms should be viewed like seat belts, helmets, and other consumer products to improve their safety.	○	○	○	○	○
America has a problem with firearm injuries as compared to other developed nations.	○	○	○	○	○
I would be open to being counseled by doctors and other health care providers about firearm safety.	○	○	○	○	○
I would be open to exploring safer storage practices with my doctor and other health care proivders.	○	○	○	○	○
I would be open to discussing how to keep children safe from firearm injury with my doctor and other health care providers.	○	○	○	○	○

I would be open to discussion with my doctor about REMOVING firearms from my home in the following situations:

	Strongly agree	Agree	Neither agree nor disagree	Disagree	Strongly disagree
If I am depressed.	○	○	○	○	○
If I am having suicidal thoughts or behaviors.	○	○	○	○	○
If my family member is depressed.	○	○	○	○	○
If my family member is having suicidal thoughts or behaviors.	○	○	○	○	○
If I am suffering from severe memory problems.	○	○	○	○	○
If my family member is suffering from severe memory problems.	○	○	○	○	○
If I am exhibiting aggressive or hostile behavior.	○	○	○	○	○
If my family member is exhibiting aggressive of hostile behavior.	○	○	○	○	○
If I am suffering from substance abuse issues.	○	○	○	○	○
If my family member is suffering from substance abuse issues.	○	○	○	○	○

I have heard someone fire a gun in person.	◯ Yes<br>◯ No
In what context?	☐ Range<br>☐ Hunting<br>☐ Home target shooting<br>☐ Competition<br>☐ Self-defense<br>☐ During a crime<br>☐ Police-related<br>☐ Unknown<br>☐ Other<br>(select all that apply)
Other	______________________________<br>(what other context?)
I have seen someone fire a gun in person.	◯ Yes<br>◯ No
In what context?	☐ Range<br>☐ Hunting<br>☐ Home target shooting<br>☐ Competition<br>☐ Self-defense<br>☐ During a crime<br>☐ Police-related<br>☐ Unknown<br>☐ Other<br>(select all that apply)
Other	______________________________<br>(what other context?)
I have fired a gun.	◯ Yes<br>◯ No
In what context?	☐ Range<br>☐ Hunting<br>☐ Home target shooting<br>☐ Competition<br>☐ Self-defense<br>☐ During a crime<br>☐ Police-related<br>☐ Other<br>(select all that apply)
Other	______________________________<br>(what other context?)

How strongly do you agree with the following?

	Strongly agree	Agree	Neither agree nor disagree	Disagree	Strongly disagree
I grew up around firearms.	◯	◯	◯	◯	◯
I feel comfortable around firearms.	◯	◯	◯	◯	◯
I know how to operate firearms.	◯	◯	◯	◯	◯
People who own firearms should have basic training, understand safety principles, and know how to use them.	◯	◯	◯	◯	◯
People should have to pass a basic safety course in order to purchase firearms.	◯	◯	◯	◯	◯
People who own firearms should have to maintain a firearm license/certification (like cars, boats, other potentially injurious consumer products).	◯	◯	◯	◯	◯
Guns don't kill people, people kill people.	◯	◯	◯	◯	◯
People who have mental health problems should not have access to firearms.	◯	◯	◯	◯	◯
People with substance abuse issues should not have access to firearms.	◯	◯	◯	◯	◯
People with felonies should not have access to firearms.	◯	◯	◯	◯	◯
Illegal immigrants should not have access to firearms.	◯	◯	◯	◯	◯
People with any criminal background should not have access to firearms.	◯	◯	◯	◯	◯
Teachers should not have access to firearms in schools.	◯	◯	◯	◯	◯

In political matters, would you say you are:

◯ Conservative
◯ Moderate
◯ Liberal
◯ Don't identify with one ideology
◯ Prefer not to say
◯ Other

Other

____

Are you a member of the NRA?

- ◯ Yes
- ◯ No
- ◯ Used to be
- ◯ Prefer not to say

How strongly do you agree with the following?

	Strongly agree	Agree	Neither agree nor disagree	Disagree	Strongly disagree
Policy makers should not infringe on the constitutional right to bear arms.	◯	◯	◯	◯	◯

Policy makers should pass legislation to limit gun access to individuals who:

	Yes	No	Not sure
Are under 21 years of age.	◯	◯	◯
Are under 18 years of age.	◯	◯	◯
Have mental health issues.	◯	◯	◯
Have substance abuse (alcohol/drug) issues.	◯	◯	◯
Have a violent criminal background.	◯	◯	◯
Have any criminal background.	◯	◯	◯
Are illegal immigrants.	◯	◯	◯
Suffer from dementia.	◯	◯	◯

How often are you afraid that you might be hurt by violence in your neighborhood?

- ◯ Always afraid
- ◯ Often afraid
- ◯ Sometimes afraid
- ◯ Rarely afraid
- ◯ Never afraid

Do you think having a gun in the house makes it a safer place to be or a more dangerous place to be?

- ◯ Safer
- ◯ More dangerous
- ◯ It depends

Please describe:

____

Have you ever been the victim of:

	Yes	No
physical violence?	◯	◯
Was a gun used (fired or not) during the incident(s)?	◯	◯
sexual violence?	◯	◯
Was a gun used (fired or not) during the incident(s)?	◯	◯
domestic violence?	◯	◯
Was a gun used (fired or not) during the incident(s)?	◯	◯
workplace violence?	◯	◯
Was a gun used (fired or not) during the incident(s)?	◯	◯

Have you ever shot an animal with a gun? ◯ Yes ◯ No

What was the context? __________________________________

Have you ever shot a person with a gun? ◯ Yes ◯ No

What was the context? __________________________________

Have you ever been shot with a gun? ◯ Yes ◯ No

What was the context? __________________________________

Have you ever been struck with a gun ("pistol whipped")? ◯ Yes ◯ No

What was the context? __________________________________

Have you ever accidentally shot yourself or another person with a gun? ◯ Yes ◯ No

What was the context? __________________________________

Have you ever needed medical treatment after accidentally injuring yourself by the "kickback", slide, hammer, or other part or action of a gun? ◯ Yes ◯ No

Have you ever been injured in another way after being threatened with a gun?<br>(Examples: injured running away from gunfire, threatened with gun to perform sexual acts, threatened with a gun to perform illegal acts, etc.)	◯ Yes<br>◯ No
What was the context?	__________________________________
Have you ever been affiliated with a gang (or a set, click, crew, etc.)?	◯ Yes<br>◯ No<br>◯ Prefer not to say
Are you currently affiliated with a gang?	◯ Yes<br>◯ No<br>◯ Prefer not to say
Which gang(s) are you affiliated with?	__________________________________
