# Supplementary material for: Patient Characteristics and Perspectives of Firearm Safety Discussions in the Emergency Department
Source: West J Emerg Med. 2021 May 19;22(3):478–87. doi: 10.5811/westjem.2021.3.49333 (PMC8203031; doi:10.5811/westjem.2021.3.49333)
Supplement: Supplementary file 2 [file wjem-22-478-s002.pdf]

Access

The following questions ask about your access to guns.

You have completed 50% of the survey. Good job!

Do you or does anyone else you live with currently own any type of gun?	◯ Yes<br>◯ No
Do you personally own a gun?	◯ Yes<br>◯ No
For how long have you owned any type of gun?	◯ 1 year or less<br>◯ Between 1 and 5 years<br>◯ Longer than 5 years
Not including yourself, how many of the people you live with, aged 18 or older, currently own any type of gun?	◯ 0 ◯ 1 ◯ 2 ◯ 3<br>◯ 4 ◯ 5 or more
What type of gun(s) do you own or have access to?	☐ Handgun (pistols, revolvers, semi-automatic revolvers, other)<br>☐ Long gun (shotgun, rifle/modern sporting rifle, other)<br>☐ Other type of gun<br>☐ I don't have access to any guns<br>(select all that apply)
What other type of gun?	__________________________________
How many TOTAL handguns do you own or have access to?	______________________<br>(enter a number)
How many TOTAL long guns do you own or have access to?	______________________<br>(enter a number)
How many TOTAL [type_gun_access_other] (other type of gun) do you own or have access to?	______________________<br>(enter a number)

If you have access to any handguns, how many are stored in each of the following places?

	0	1	2	3	4	5 or more
Home	◯	◯	◯	◯	◯	◯
Garage at home	◯	◯	◯	◯	◯	◯
Another building at home (like a barn or storage shed)	◯	◯	◯	◯	◯	◯
Car or other motor vehicle	◯	◯	◯	◯	◯	◯
Work	◯	◯	◯	◯	◯	◯
Someplace else	◯	◯	◯	◯	◯	◯

If someplace else, where?

____

For what reasons do you have access to/own a handgun? (select ALL that apply)

☐ For protection against strangers.
☐ For protection against people I know.
☐ For protection against animals.
☐ For hunting.
☐ For other sporting use.
☐ For a collection/hobby.
☐ For some other reason.
(select all that apply)

If for another reason, for what?

____

What is the PRIMARY reason you have access to/own a handgun? (select the ONE best answer)

◯ For protection against strangers.
◯ For protection against people I know.
◯ For protection against animals.
◯ For hunting.
◯ For other sporting use.
◯ For a collection/hobby.
◯ For some other reason.

If for another reason, for what?

____

Is any handgun stored loaded and unlocked?

◯ Yes
◯ No

How is/are the handgun(s) usually stored?

◯ Loaded and unlocked
◯ Loaded and locked
◯ Unloaded and unlocked
◯ Unloaded and locked

If you have access to any long guns, how many are stored in each of the following places?	0	1	2	3	4	5 or more
Home	○	○	○	○	○	○
Garage at home	○	○	○	○	○	○
Another building at home (like a barn or storage shed)	○	○	○	○	○	○
Car or other motor vehicle	○	○	○	○	○	○
Work	○	○	○	○	○	○
Someplace else	○	○	○	○	○	○

If someplace else, where?

____

For what reasons do you have access to/own a long gun? (select ALL that apply)

- ☐ For protection against strangers.
- ☐ For protection against people I know.
- ☐ For protection against animals.
- ☐ For hunting.
- ☐ For other sporting use.
- ☐ For a collection/hobby.
- ☐ For some other reason.

(select all that apply)

If for another reason, for what?

____

What is the PRIMARY reason you have access to/own a long gun? (select the ONE best answer)

- ○ For protection against strangers.
- ○ For protection against people I know.
- ○ For protection against animals.
- ○ For hunting.
- ○ For other sporting use.
- ○ For a collection/hobby.
- ○ For some other reason.

If for another reason, for what?

____

Is any long gun stored loaded and unlocked?

- ○ Yes
- ○ No

How is/are the long gun(s) usually stored?

- ○ Loaded and unlocked
- ○ Loaded and locked
- ○ Unloaded and unlocked
- ○ Unloaded and locked

If you have access to another type of gun, how many guns are stored in each of the following places?

	0	1	2	3	4	5 or more
Home	◯	◯	◯	◯	◯	◯
Garage at home	◯	◯	◯	◯	◯	◯
Another building at home (like a barn or shed)	◯	◯	◯	◯	◯	◯
Car or other motor vehicle	◯	◯	◯	◯	◯	◯
Work	◯	◯	◯	◯	◯	◯
Someplace else	◯	◯	◯	◯	◯	◯

If someplace else, where?

____

For what reasons do you have access to/own a [type_gun_access_other] gun? (select ALL that apply)

- ☐ For protection against strangers.
- ☐ For protection against people I know.
- ☐ For protection against animals.
- ☐ For hunting.
- ☐ For other sporting use.
- ☐ For a collection/hobby.
- ☐ For some other reason.

(select all that apply)

If for another reason, for what?

____

What is the primary reason you have access to/own a [type_gun_access_other] gun? (select the ONE best answer)

- ◯ For protection against strangers.
- ◯ For protection against people I know.
- ◯ For protection against animals.
- ◯ For hunting.
- ◯ For other sporting use.
- ◯ For a collection/hobby.
- ◯ For some other reason.

If for another reason, for what?

____

Is any [type_gun_access_other] gun stored loaded and unlocked?

- ◯ Yes
- ◯ No

How is/are the [type_gun_access_other] gun(s) usually stored?

- ◯ Loaded and unlocked
- ◯ Loaded and locked
- ◯ Unloaded and unlocked
- ◯ Unloaded and locked

You have completed 75% of the survey.

Do you have any of the following for any of your guns?

- ☐ Cable lock
- ☐ Trigger lock
- ☐ Intrinsic lock (built into the gun)
- ☐ Lock box / gun safe
- ☐ Other locked storage container
- ☐ No / none

(select all that apply)

Please describe:

____

In the past 12 months, have you had any firearms stolen from you?

- ◯ Yes
- ◯ No

Where was the gun(s) stolen from?

- ☐ My home
- ☐ My garage at my home
- ☐ Another building at my home
- ☐ My car or other motor vehicle
- ☐ My work
- ☐ Someplace else

(select all that apply)

Please describe where the firearm was stolen from:

____

How much do you agree with the following?

	Strongly agree	Agree	Neither agree nor disagree	Disagree	Strongly disagree
My/our gun makes me feel safer.	◯	◯	◯	◯	◯
I don't want only the bad guys to have guns.	◯	◯	◯	◯	◯
It is my duty as an American to have a gun(s).	◯	◯	◯	◯	◯
I worry about my/our gun being used against me.	◯	◯	◯	◯	◯
I worry about my partner intentionally injuring me/others with my/our gun.	◯	◯	◯	◯	◯
I worry about my partner unintentionally injuring me/others with my/our gun.	◯	◯	◯	◯	◯
I worry about children in my home intentionally injuring me/others with my/our gun.	◯	◯	◯	◯	◯
I worry about children in my home unintentionally injuring me/others with my/our gun.	◯	◯	◯	◯	◯
I worry I will accidentally injure a family member with my/our gun	◯	◯	◯	◯	◯
I worry I will accidentally harm myself with my/our gun.	◯	◯	◯	◯	◯
I worry I will intentionally harm myself or others with my/our gun.	◯	◯	◯	◯	◯
If I seek help for mental health or substance abuse issues, I worry my/our gun will be taken away from me/us.	◯	◯	◯	◯	◯
Children in my home do not know where firearm(s) are stored.	◯	◯	◯	◯	◯
Children in my home know where firearm(s) are located, but do not have access to it/them (locked in a safe or lock box)	◯	◯	◯	◯	◯
I feel confident I will not injure myself or my family with my/our firearm.	◯	◯	◯	◯	◯

I took a course on gun safety.

◯ Yes
◯ No

What kind of course?

- [ ] Range-based
- [ ] NRA-sponsored
- [ ] Internet-based
- [ ] Through a friend
- [ ] Military-related
- [ ] Other

(select all that apply)

Please describe the type of course you took: ____

How much do you agree with the following?

	Strongly agree	Agree	Neither agree nor disagree	Disagree	Strongly disagree
I feel safe at home.	○	○	○	○	○
I have never felt the need to own a gun.	○	○	○	○	○
I have not been around guns.	○	○	○	○	○
If I had a gun, I would feel safer.	○	○	○	○	○
I worry about being shot by a stranger in my community.	○	○	○	○	○
I worry about being shot by a co-worker/acquaintance.	○	○	○	○	○
I worry about being shot by a partner/family member.	○	○	○	○	○
I feel that gun owners have a responsibility to be well-educated about gun safety.	○	○	○	○	○
I find it difficult to discuss guns with people that do not own guns.	○	○	○	○	○
I find it difficult to discuss guns with people that own guns.	○	○	○	○	○
